# Characterization of the semen, gut, and urine microbiota in patients with different semen abnormalities

**DOI:** 10.3389/fmicb.2023.1182320

**Published:** 2023-05-24

**Authors:** Tingshuai Cao, Shangren Wang, Yang Pan, Feng Guo, Bin Wu, Yingchun Zhang, Yujie Wang, Jiaqing Tian, Qingfei Xing, Xiaoqiang Liu

**Affiliations:** ^1^Department of Urology, Tianjin Medical University General Hospital, Tianjin, China; ^2^Department of Urology, Central Hospital Affiliated to Shandong First Medical University, Jinan, China; ^3^Center for Reproductive Medicine, Central Hospital Affiliated to Shandong First Medical University, Jinan, China

**Keywords:** microbiota, semen, urine, gut, 16S rRNA, semen abnormalities

## Abstract

**Introduction:**

Semen quality is decreasing worldwide, leading to increased male infertility. This study analyzed the microbiota of the gut, semen, and urine in individuals with semen abnormalities to identify potential probiotics and pathogenic bacteria that affect semen parameters and help develop new methods for the diagnosis and treatment of patients with semen abnormalities.

**Methods:**

We recruited 12 individuals with normal semen parameters (control group), 12 with asthenospermia but no semen hyperviscosity (Group_1), 6 with oligospermia (Group_2), 9 with severe oligospermia or azoospermia (Group_3), and 14 with semen hyperviscosity only (Group_4). The semen, gut, and urine microbiota were examined by analyzing the 16S ribosomal RNA gene sequence using next-generation sequencing.

**Results:**

The gut microbes were clustered into the highest number of operational taxonomic units, followed by urine and semen. Furthermore, the α-diversity of gut microbes was highest and significantly different from that of urine and semen microbiota. The microbiota of the gut, urine, and semen were all significantly different from each other in terms of β-diversity. The gut abundance of *Collinsella* was significantly reduced in groups 1, 3, and 4. Furthermore, the gut abundance of *Bifidobacterium* and *Blautia* was significantly decreased in Group_1, while that of *Bacteroides* was significantly increased in Group_3. The abundance of *Staphylococcus* was significantly increased in the semen of groups 1 and 4. Finally, *Lactobacillus* abundance was significantly reduced in the urine of groups 2 and 4.

**Discussion:**

This study comprehensively describes the differences in intestinal and genitourinary tract microbiota between healthy individuals and those with abnormal semen parameters. Furthermore, our study identified *Collinsella*, *Bifidobacterium*, *Blautia*, and *Lactobacillus* as potential probiotics. Finally, the study identified *Bacteroides* in the gut and *Staphylococcus* in semen as potential pathogenic bacteria. Our study lays the foundation of a new approach to the diagnosis and treatment of male infertility.

## 1. Introduction

Studies have shown that semen quality is severely declined worldwide (Carlsen et al., 1992; Levine et al., 2017; Sengupta et al., 2018), which is associated with increased male infertility (Said et al., 2004; Dohle et al., 2005). However, the specific causes for this decline require further exploration and research. Genitourinary tract infections are thought to be involved in reducing semen quality (Jequier, 1985; Urata et al., 2001; Oghbaei et al., 2020); however, the role of specific bacteria might have been underestimated as these previous studies have used traditional bacterial culture methods, which can identify less than 2% of all known bacteria (Wade, 2002). Fortunately, 16S ribosomal RNA (rRNA) gene sequencing can provide a detailed and reliable classification of microbes present in various body compartments.

Previous studies have shown that the gut microbiota or its products can affect multiple organs other than the gut, including the male reproductive system. Gut microbiota transplantation has beneficial effects on pediatric autism (Kang et al., 2017, 2019) and ameliorates fertility in male mice (Zhang et al., 2021; Hao et al., 2022a,b). Gut flora can also influence the permeability of the blood–brain barrier (BBB) (Braniste et al., 2014; Hoyles et al., 2021). The bidirectional regulation of the microbiome-gut-brain axis plays an important role in regulating immune and metabolic processes (Zhang et al., 2015), promoting androgen production, and regulating gonadal development and testicular immunity (Cai et al., 2022). Gut microbiota dysbiosis can lead to increased permeability of the blood-testis barrier (BTB) and affect serum follicle-stimulating hormone (FSH), luteinizing hormone (LH), and testosterone levels in mice (Al-Asmakh et al., 2014). Perturbations in the gut microbiota impair testicular functions in mice and rats and reduce semen utilization rate in Duroc boars (Guo et al., 2020; Zhao et al., 2021; Liu et al., 2022). Amino acids play an important role in regulating reproductive processes such as spermatogenesis (Dai et al., 2015; Zhao et al., 2021). Numerous studies have shown that gut bacteria play an important role in amino acid metabolism and recycling (Fuller and Reeds, 1998; Dai et al., 2011; Neis et al., 2015; Dodd et al., 2017; Liu et al., 2020; Agus et al., 2021; Zhao et al., 2021). Therefore, it is essential to study the relationship between gut microbiota and semen quality. Furthermore, several studies have demonstrated that the normal human semen (Rodin et al., 2003; Hou et al., 2013; Baud et al., 2019; Yang et al., 2020; Lundy et al., 2021) and testicular microenvironment is non-sterile (Alfano et al., 2018; Molina et al., 2021) and testicular/epididymal microbiome is present in the human semen (Lundy et al., 2021). Studies have reported that the paternal semen microbiome can influence the health of the offspring (Rando and Simmons, 2015; Feng and Liu, 2022). The offspring of the high-protein diet-fed male rats exhibit enhanced production of short-chain fatty acids (SFCAs) and increased abundance of *Bifidobacterium* in the gut (Chleilat et al., 2021). This finding suggests that the health of the father can influence the health of the offspring. An ecologically normal microbiota in semen is likely necessary for normal sperm production and function, and the good health of the offspring. However, little is known about the effects of semen, gut, and urine microbiomes on semen parameters. Herein, we performed 16S rRNA gene sequencing to identify the specific flora of the gut, semen, and urine. Furthermore, we also tried to identify the potentially pathogenic bacteria that might be associated with abnormal semen parameters. The outcomes of this study lay the groundwork for a new approach to diagnosing and treating men with reduced semen quality.

## 2. Materials and methods

### 2.1. Study participants

Fifty-three participants from the Central Hospital Affiliated to Shandong First Medical University were recruited. The participants were divided into the healthy control group and four abnormal semen groups. The healthy control group had normal semen parameters. We recruited 12 individuals with normal semen parameters (control group), 12 with asthenospermia (proportion of forward motile sperm <32%) but no semen hyperviscosity (Group_1), 6 with oligospermia (sperm concentration < 15 million/mL) (Group_2), 9 with severe oligospermia or azoospermia (sperm concentration < 2 million/mL) (Group_3), and 14 with semen hyperviscosity only (Semen still forms a thread of more than 2 cm after 1 h of liquefaction) (Group_4). Semen abnormalities were defined according to the fifth edition of the World Health Organization. All participants met the following requirements: no antibiotic use within 3 months, no history of genitourinary surgery within 1 year, no long-term exposure to toxic and harmful substances, no history of tumor, no testosterone use within 2 years, no history of hypertension, and no history of diabetes. All individuals volunteered to participate in this clinical study and signed an informed consent form.

### 2.2. Ethics statement

The Ethics Committee of Shandong First Medical University approved this study with ethics approval no. (R202203030053).

### 2.3. Sample collection

All participants abstained from sex and masturbation for 2–7 days before semen collection. The detailed aseptic procedure for sample collection was explained to all participants. Before semen collection, all participants washed their hands thrice with soap. The penis, glans, and coronal sulcus were washed three times with soap and dried with sterile gauze. Finally, semen was collected directly into a sterile container. Similarly, before collecting urine, the penis, glans, and coronal sulcus were washed three times with soap and dried with sterile gauze. Subsequently, 10 mL of midstream urine was collected in a sterile container. Approximately 1 g of fresh feces was collected with a sterile spoon in a 5 mL sterile fecal collection container. The collected fecal and urine samples were stored at −80°C. After semen collection, a routine semen examination was performed, and the remaining semen samples were transferred to sterile cryotubes and stored at −80°C. All participants had their samples collected within 24 h of participating in the study. The order was semen, followed by urine and finally feces. This order was followed by all participants. The collection of all samples was done in the hospital.

### 2.4. DNA extraction

DNA was extracted from the three samples using the PowerSoil DNA Isolation Kit (MoBio Laboratories, Carlsbad, CA, United States) and Omega Stool DNA Kit following the manufacturer’s instructions. Samples that passed the DNA quality and concentration tests were stored at −80°C for subsequent experiments.

### 2.5. 16S ribosomal RNA gene sequencing

The primers 338F (5′-ACTCCTACGGGAGGCAGCAG-3′) and 806R (5′-GGACTACNNGGGTATCTAAT-3′) (Munyaka et al., 2015) were used to amplify the V3-V4 hypervariable region of the bacterial 16S rRNA gene. An 8 bp barcode sequence was added at the 5′ end of each upstream and downstream primers to distinguish between different samples. Amplicons were separated on 2% agarose gels, purified using the AxyPrep DNA Gel Extraction Kit according to the manufacturer’s instructions, and quantified using QuantiFluor-ST. The purified amplicons were pooled in equimolar concentrations and paired-end sequenced (2 × 250) on the Illumina platform according to standard protocols. PCR reaction mixture (25 μL) contained 12.5 μL 2xTaq Plus Master Mix, 3 μL BSA (2 ng/μL), 1 μL Forward Primer (5 μM), 1 μL Reverse Primer (5 μM), 2 μL template DNA, and 5.5 μL ddH_2_O. Reaction parameters were as follows: pre-denaturation at 95°C for 5 min followed by 28 cycles of denaturation at 95°C for 45 s, annealing at 55°C for 50 s, and extension at 72°C for 45 s, and a final step of extension at 72°C for 10 min. The PCR products were detected by 1% agarose gel electrophoresis to determine the size of the amplified target bands, which were then purified using the Agencourt AMPure XP Nucleic Acid Purification Kit.

### 2.6. MiSeq sequencing

The PCR products were used to construct a microbial diversity sequencing library and paired-end sequencing was performed. The raw data were uploaded to the SRA database of NCBI (accession number: PRJNA900680).

### 2.7. Data analysis and processing

The raw data were split using the QIIME1 (v1.8.0) software based on the barcode sequences, and the data were filtered and spliced using the Pear (v0.9.6) software. To obtain high-quality clean reads: (a) reads containing >10% of unknown nucleotides (N) were removed and (b) reads containing <80% of bases with quality (Q-value) >20 were filtered out. The minimum overlap was set to 10 bp and the mismatch rate was set to 1%. After splicing, sequences less than 230 bp in length were removed using the Vsearch (v2.7.1) software, and chimeric sequences were removed by comparing with the Gold Database using the UCHIME method. Effective tags were clustered into operational taxonomic units (OTUs) with ≥ 97% similarity using the MOTHUR pipeline. The tag sequence with the highest abundance was selected as the representative sequence for each cluster. OTU clustering was performed using the Vsearch (v2.7.1) software. UPARSE algorithm was used to obtain quality sequences with a sequence similarity threshold of 97% (Edgar, 2013). The Ribosomal Database Project (RDP) Classifier tool was used to classify all sequences into different taxonomic groups against the SILVA database (release 128) (Cole et al., 2009). Representative sequences were classified into organisms based on the naïve Bayesian model using the RDP classifier (V.2.2) based on Greengenes (V.gg_13_5). QIIME (v1.8.0) was used to calculate richness and diversity indices based on the OTU information. To compare the composition and structure of microbial communities in different samples, a heat map of the top 20 OTUs was generated using Mothur (Jami et al., 2013). Non-metric multidimensional scaling (NMDS) was performed and data was plotted using QIIME (v1.8.0) and ggplot2 3.3.2. Stamp analysis was performed using the STAMP 2.1.3 software and comparisons between the two groups were made using Welch’s *t*-test.

### 2.8. Statistical analysis

Continuous data were tested for normal distribution using the Shapiro–Wilk test. If normally distributed, the data were expressed as mean (standard deviation), and comparisons between groups were performed using the analysis of variance (ANOVA) while Bonferroni’s method was used for two-way comparisons. The non-normally distributed data were expressed as median with interquartile ranges (25th–75th percentiles) (IQR), and the overall comparison between groups was performed using the Kruskal-Wallis test. Two-way comparisons were performed using the Kruskal-Wallis one-way ANOVA. Categorical data were expressed as numbers and proportions, and the overall comparison between groups was performed using the corrected chi-square test. Differences in the abundance of various bacterial species were analyzed using Welch’s t-test. The relationship between selected differentially abundant genera and abnormal semen and demographic parameters was analyzed using a generalized linear model. When analyzing the gut microbiota sequencing data, we performed a two-tailed Wilcoxon rank-sum test using the R Project. A value of *p* < 0.05 was considered statistically significant.

## 3. Results

### 3.1. Demographic characteristics and semen parameters

To minimize, as much as possible, the effects of relevant confounding factors, we selected individuals with similar age, body mass index (BMI), smoking status, and alcohol consumption in each study group (Table 1).

**Table 1:** Demographic characteristics and semen parameters of the study participants.

	Control	Group 1	Group 2	Group 3	Group 4
Number	12	12	6	9	14
Age, year, median (IQR)	30.75 (28.5–34.0)	33.67 (30–36)	30.00 (26.5–34.5)	34.22 (28.5–36.5)	30.29 (27–34)
BMI, kg/m^2^, mean (SD)	26.03 (2.28)	25.21 (2.56)	23.80 (3.24)	24.03 (4.21)	26.43 (3.79)
Smoking status, *n* (%)					
Yes	6 (50%)	3 (25%)	5 (83%)	3 (33%)	5 (36%)
No	6 (50%)	9 (75%)	1 (17%)	6 (67%)	9 (64%)
Alcohol use, *n* (%)					
Yes	7 (58%)	5 (42%)	1 (17%)	3 (33%)	4 (29%)
No	5 (42%)	7 (58%)	5 (83%)	6 (67%)	10 (71%)
Semen volume, mL, mean (SD)	3.94 (1.84)	3.69 (1.03)	3.95 (0.86)	3.81 (1.18)	3.79 (1.17)
Semen pH, median (IQR)	7.40 (7.33–7.50)	7.22 (7.20–7.40)*	7.40 (7.20–7.52)	7.23 (6.95–7.5)	7.44 (7.38–7.5)
Sperm concentration, million/mL, mean (SD)	77.37 (45.78)	53.05 (33.43)	12.62 (1.87)*	NA	86.15 (50.35)
Progressive motility in percentage, mean (SD)	51.29 (11.20)	15.69 (6.56)**	21.75 (10.39)**	NA	46.44 (7.54)
Semen hyperviscosity, *n* (%)	0 (0%)	0 (0%)	1 (17%)	2 (11%)	14 (100%)

IQR, interquartile range; BMI, body mass index; SD, standard deviation. *p < 0.05 and **p < 0.001 compared to the control group.

### 3.2. Microbiota composition of the gut, semen, and urine

**Figure 1:**
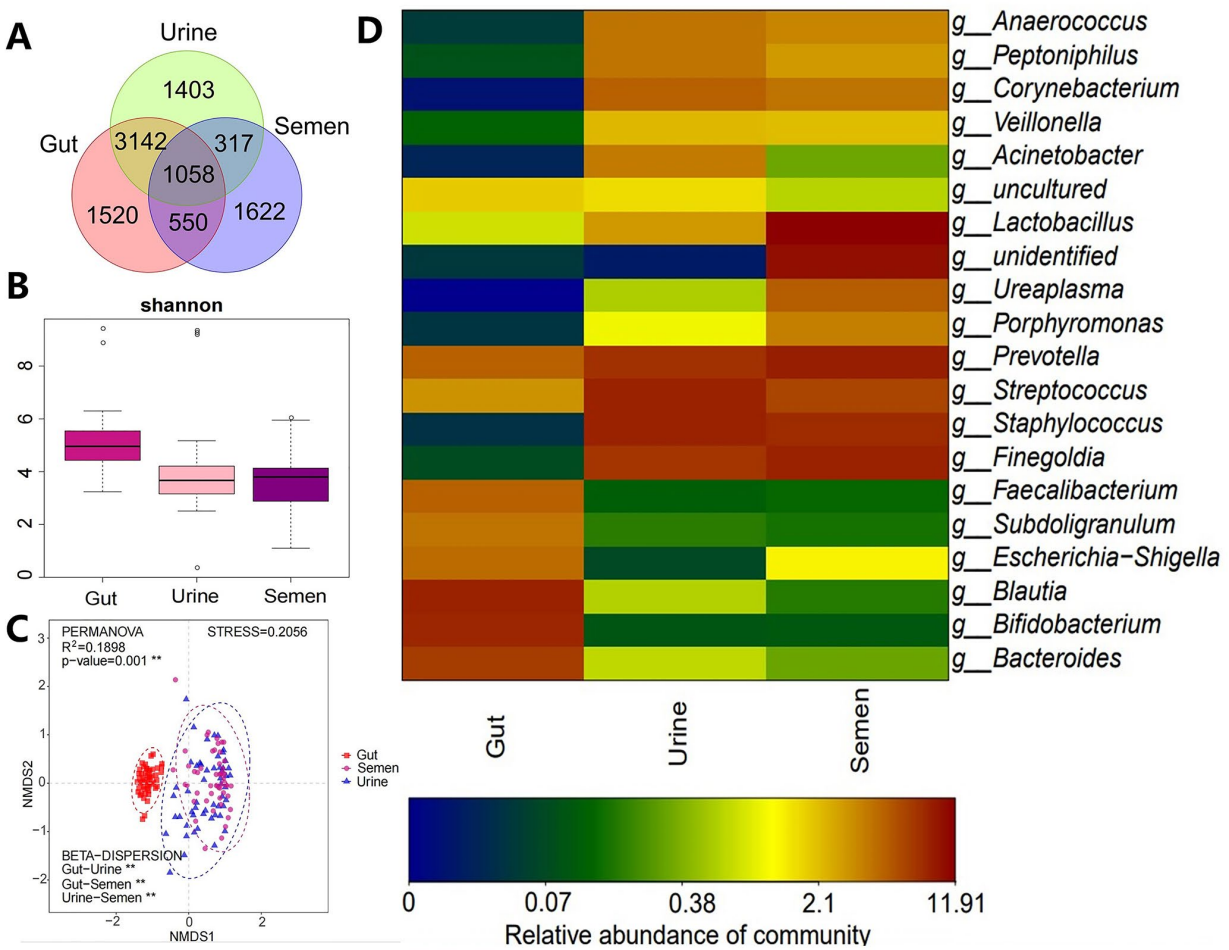
**(A)** Veen diagram showing OTUs enriched in the gut, urine, and semen. **(B)** Shannon index was used to estimate the *α*-diversity. **(C)** Graph showing β-diversity. **p < 0.001. **(D)** Heatmap showing the relative abundance of the topmost differentially abundant OTUs in the gut, urine, and semen.

One participant of Group_3 failed to provide the stool sample while the genomic DNA extraction and amplification from the semen of another participant was unsuccessful, thus, both of these samples were excluded and 157 samples were finally analyzed. These 157 samples yielded 12,393,226 raw sequences and 11,140,437 clean sequences, of which, high-quality sequences were mainly distributed in the range of 400–440 bp. The total reads were clustered into 9,612 OTUs, which included 6,270 OTUs in the gut, 5,920 in urine, and 3,547 in semen, for a total of 1,058 OTUs for all three kinds of samples (Figure 1A). The α-diversity of the gut microbes was higher and significantly different from that of urine and semen microbiota; however, no significant difference was observed between urine and semen (Figure 1B). The gut, urine, and semen microbiota were all significantly different from each other in terms of β-diversity (Figure 1C). The phylum of the top four gut and urine are the same including *Firmicutes*, *Actinobacteriota*, *Bacteroidetes*, and *Proteobacteria* (Supplementary Figure S1). *Firmicutes*, *Bacteroidetes*, *unidentified*, and *Actinobacteriota* are the four main phylum in semen (Supplementary Figure S1). At the genus level, *Bifidobacterium*, *Blautia*, *Bacteroides*, *Faecalibacterium*, and *Prevotella* were predominant in the gut, *Staphylococcus*, *Streptococcus*, *Prevotella*, *Finegoldia*, and *Corynebacterium* were predominant in urine, and *Lactobacillus*, *Prevotella*, *Finegoldia*, *Staphylococcus*, *Streptococcus*, *Ureaplasma*, and other unidentified bacteria were predominant in semen (Figure 1D).

**Figure 2:**
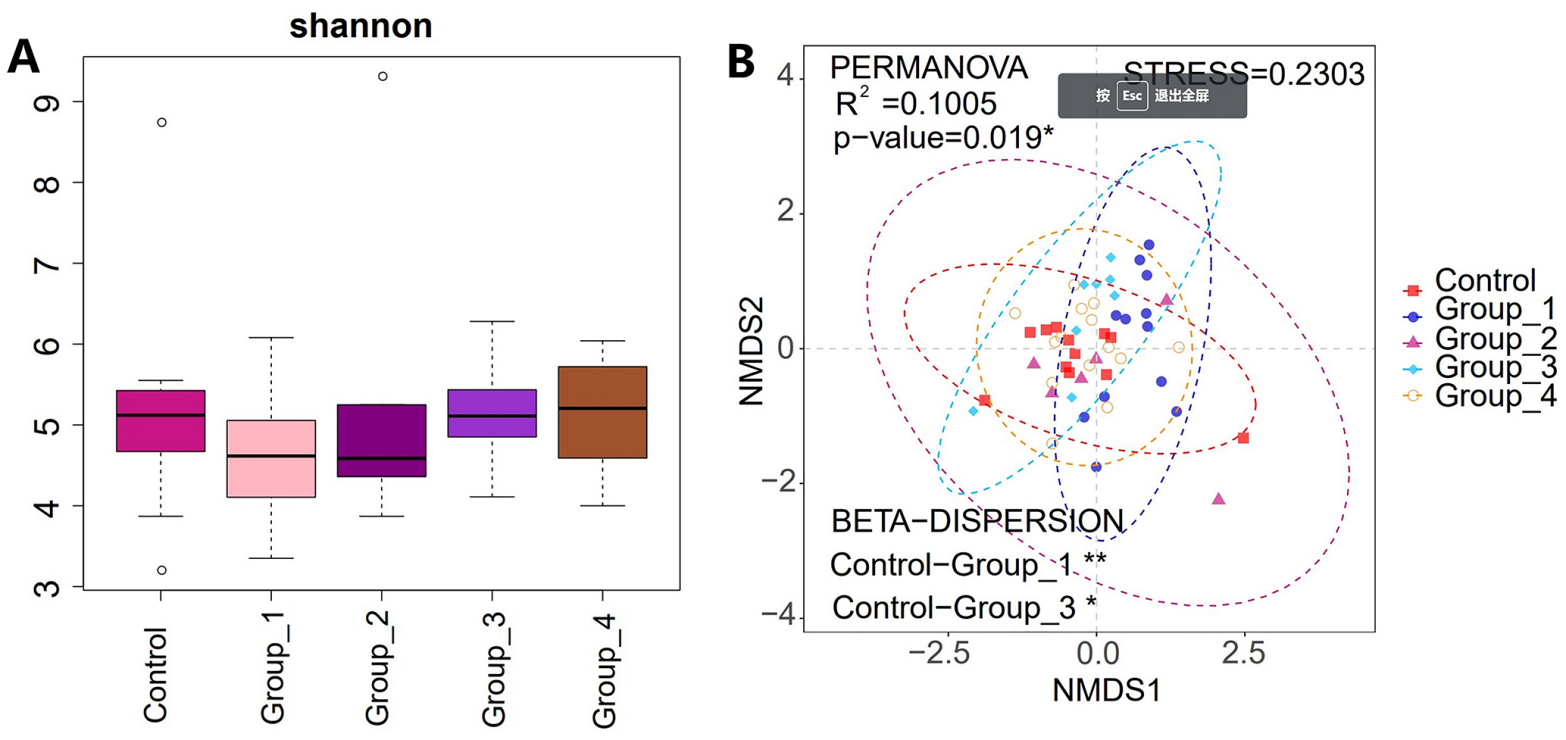
**(A)** Shannon index was used to estimate the *α*-diversity of the gut microbiota. **(B)** Graph showing *β*-diversity of the gut microbes.

**Figure 3:**
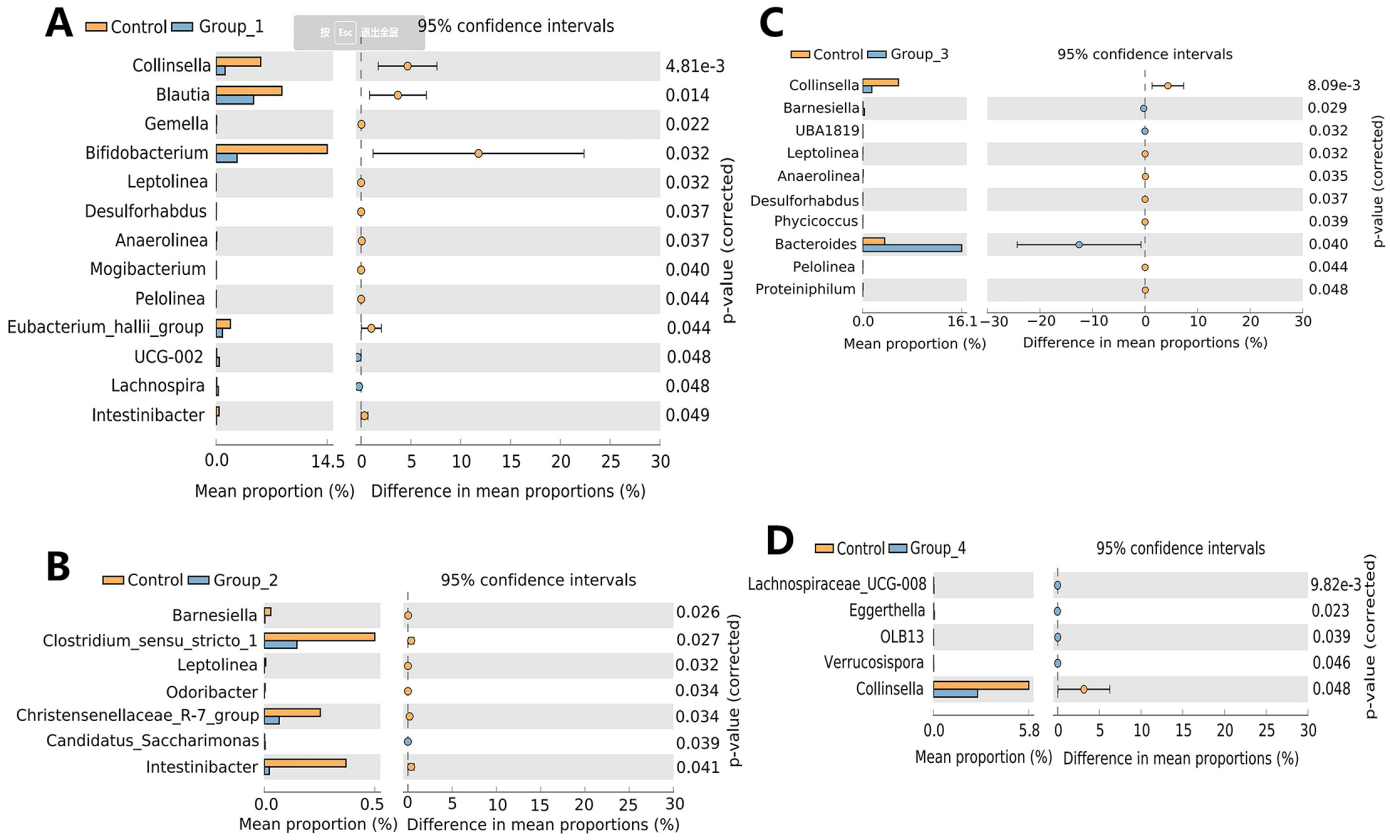
Stamp analysis showing differences in the gut microbiota between the control group, Group_1 **(A)**, Group_2 **(B)**, Group_3 **(C)**, and Group_4 **(D)**.

The α-diversity of the gut microbiota was not significantly different between the control and abnormal semen groups (Figure 2A). While the β-diversity of the control group was significantly different from that of groups 1 and 3 only (Figure 2B). In Group_1, the gut abundance of *Collinsella*, *Blautia*, and *Bifidobacterium* was significantly decreased (Figure 3A). In Group_2, the gut abundance of *Clostridium sensu stricto 1*, the *Christensenellaceae* R-7 group, and *Intestinibacter* was significantly decreased (Figure 3B). In Group_3, there was a significant decrease in the gut abundance of *Collinsella* and a significant increase in that of *Bacteroides* (Figure 3C). In Group_4, there was a significant decrease in the gut *Collinsella* abundance (Figure 3D).

**Figure 4:**
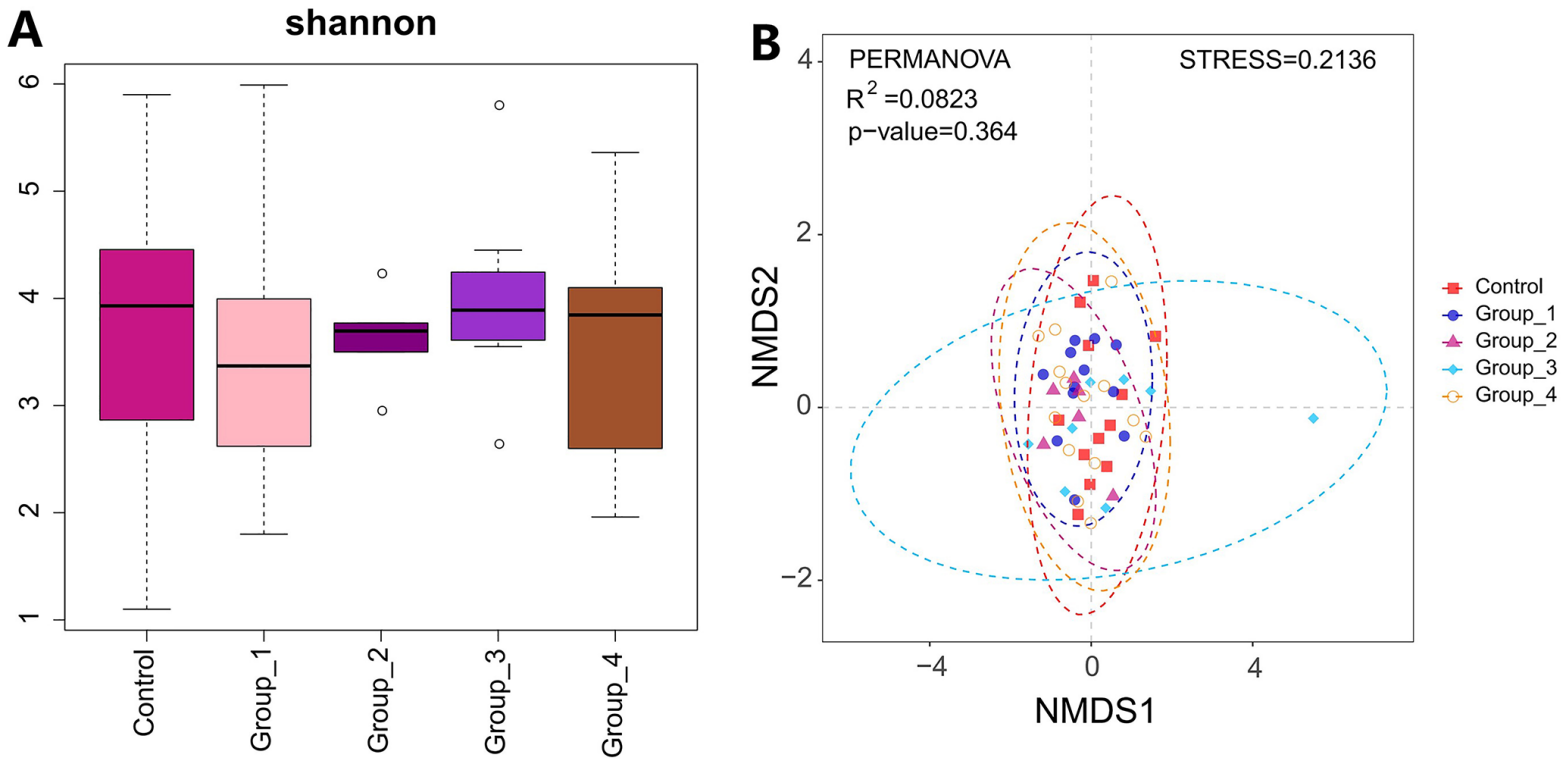
**(A)** Shannon index was used to estimate the *α*-diversity of semen microbiota. **(B)** Graph showing *β*-diversity of semen microbiota.

**Figure 5:**
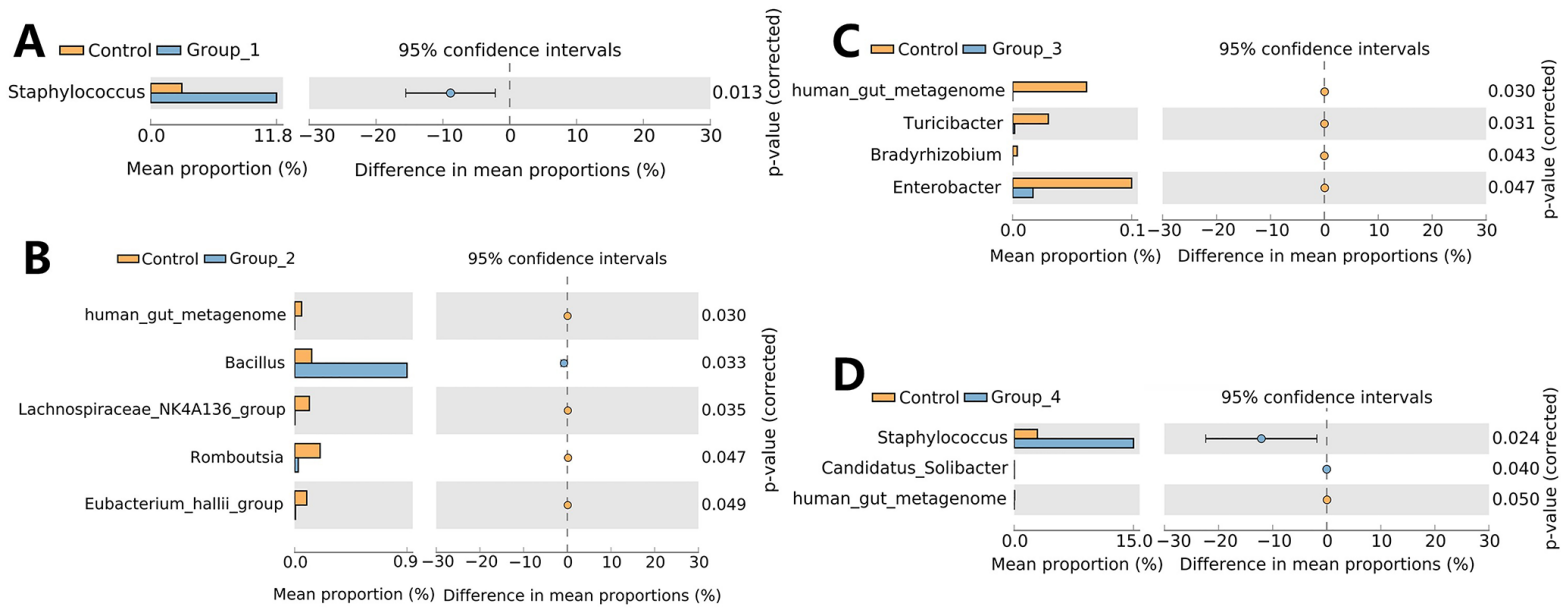
Stamp analysis showing differences in semen microbiota between the control group, Group_1 **(A)**, Group_2 **(B)**, Group_3 **(C)**, and Group_4 **(D)** at the genus level.

The α- and β-diversities of the seminal microbiota were not significantly different between the control and abnormal semen groups (Figure 4). However, groups 1 and 4 showed a significant increase in seminal *Staphylococcus* abundance (Figures 5A,D).

**Figure 6:**
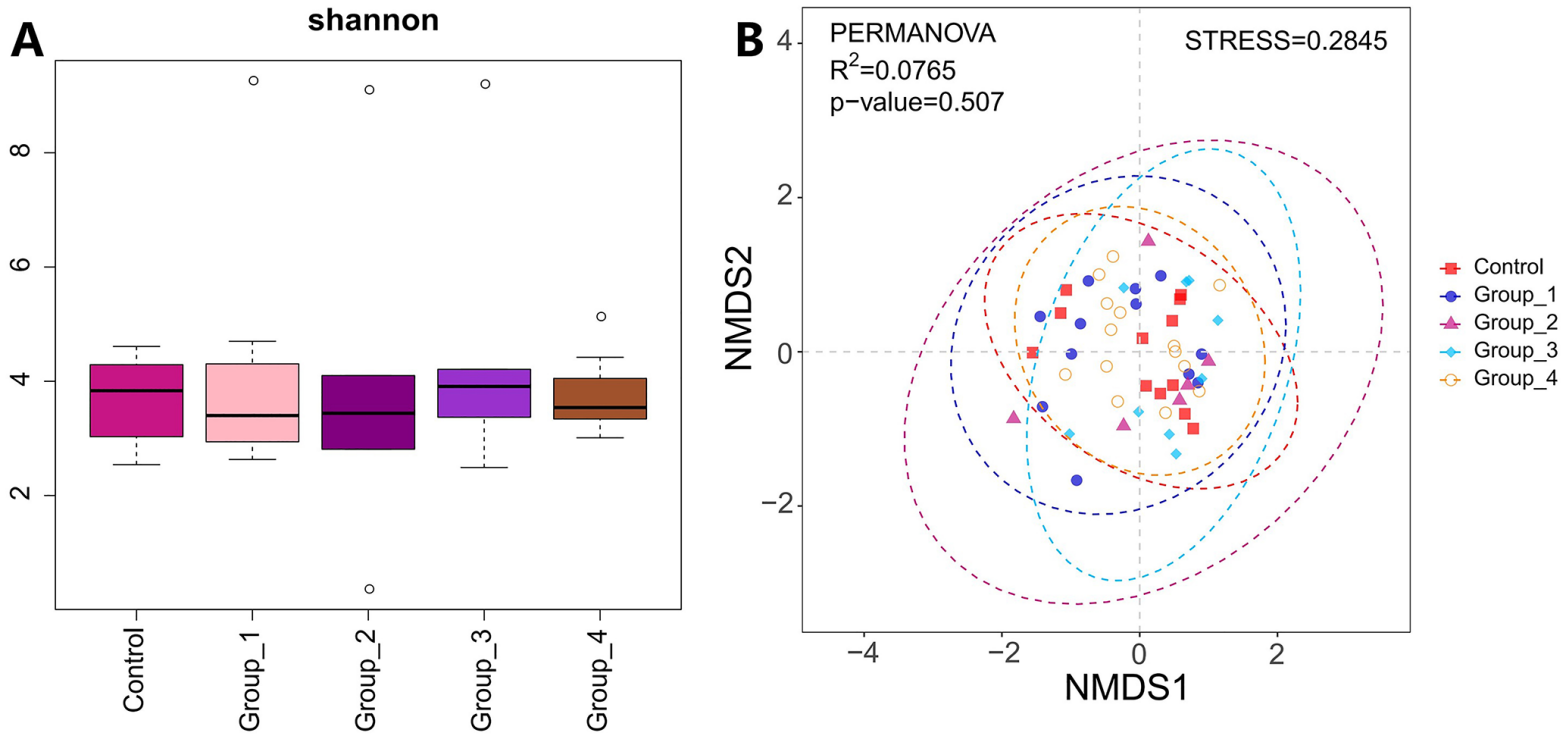
**(A)** Shannon index was used to estimate the *α*-diversity of microbes present in urine. **(B)** Graph showing *β*-diversity of urine microbiota.

**Figure 7:**
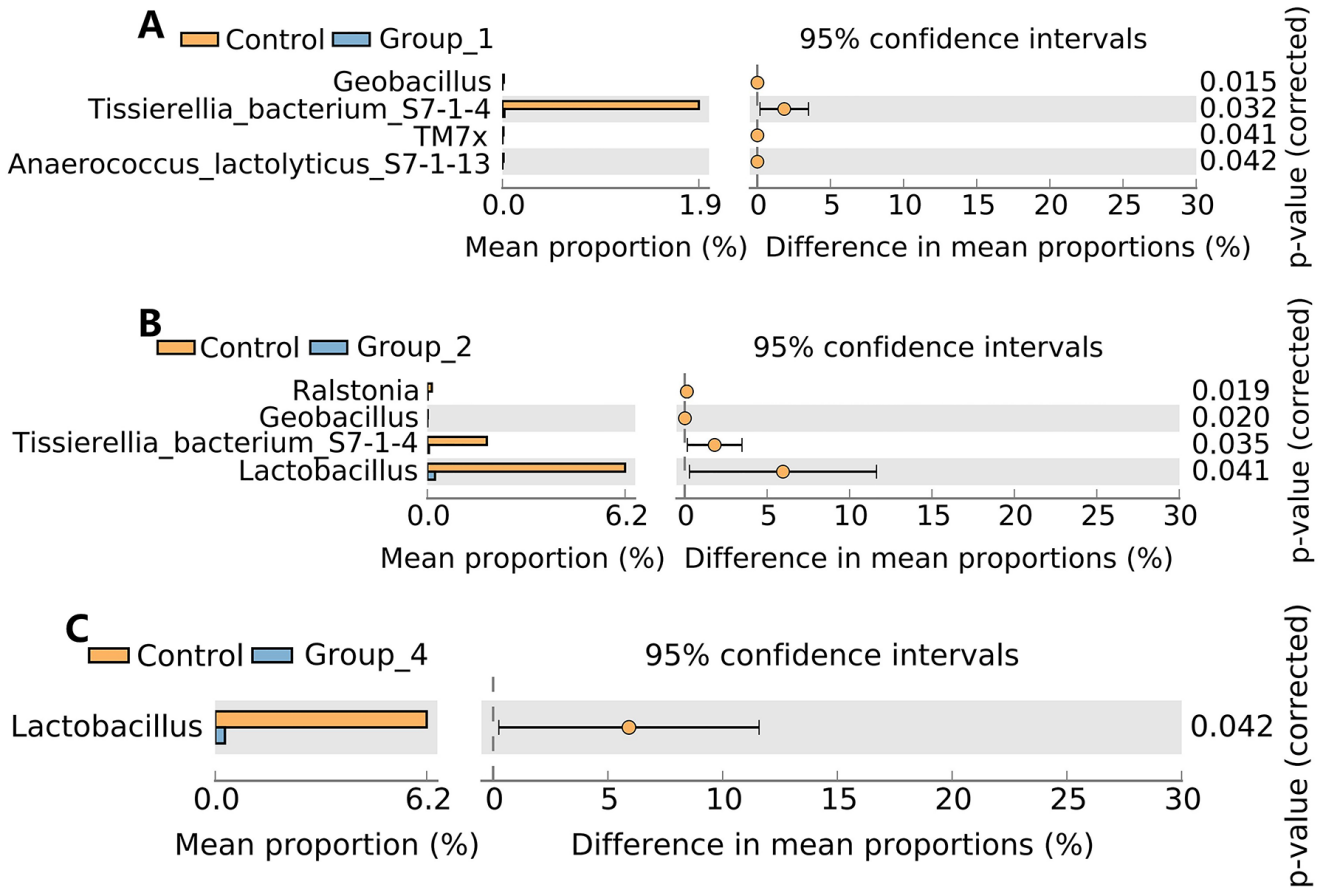
Stamp analysis showing differences in urine microbiota between the control group, Group_1 **(A)**, Group_2 **(B)**, and Group_4 **(C)** at the genus level. There was no significant difference between the control group and Group_3.

Regarding urinary microbiota, no significant difference in the α- and β-diversities was observed between the control and abnormal semen groups (Figure 6). In Group_1, the urinary abundance of the *Tissierellia* bacterium S7-1-4 was significantly reduced (Figure 7A). Group_2 showed a significant decrease in the urinary abundance of *Lactobacillus* and the *Tissierellia* bacterium S7-1-4 (Figure 7B). There were no significant differences between Group_3 and the control group. Group_4 exhibited a significant decrease in urinary *Lactobacillus* abundance (Figure 7C).

### 3.3. Generalized linear models for selected genera and key clinical characteristics

**Figure 8:**
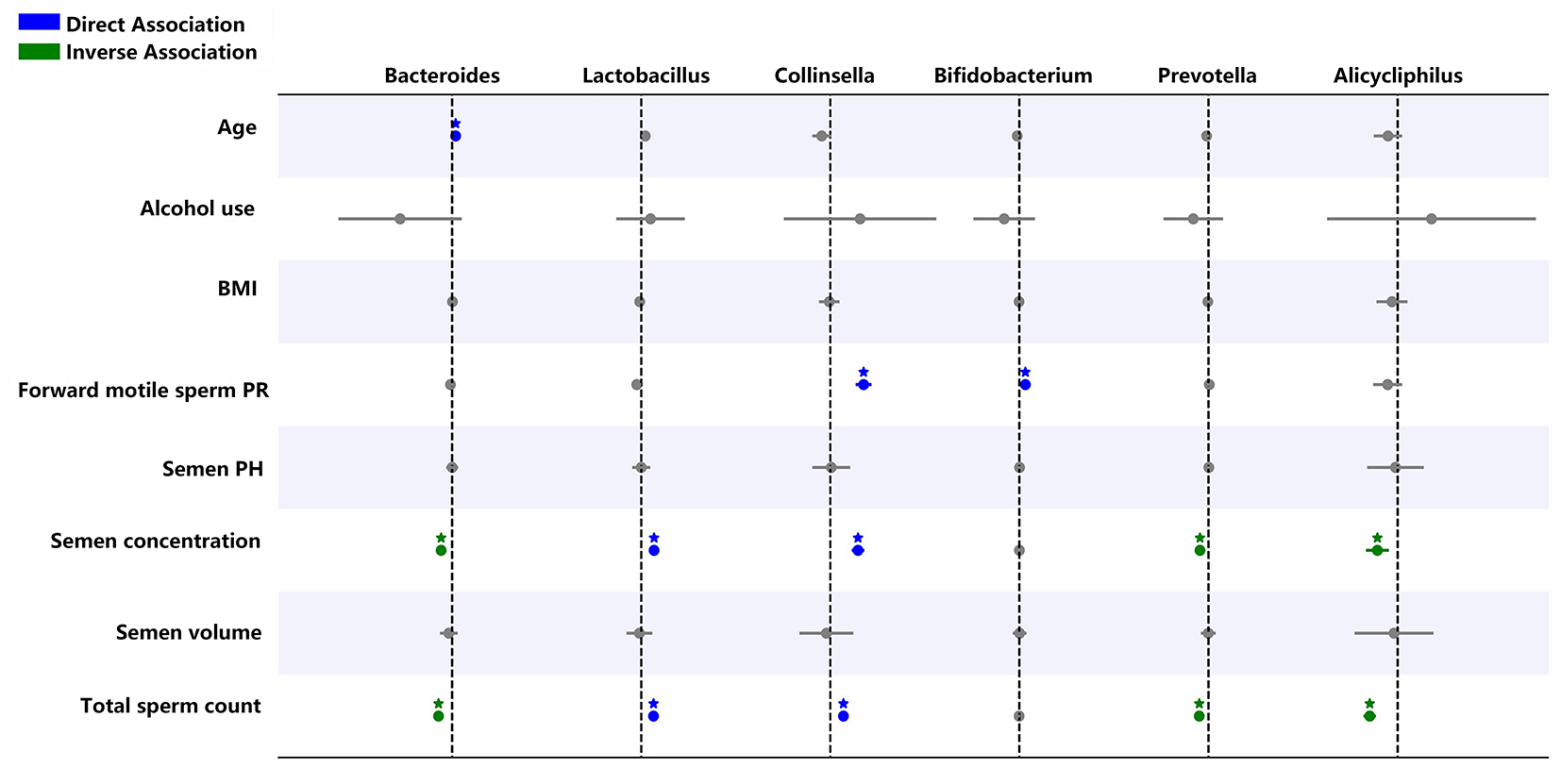
Generalized linear regression models showing the relationship between selected genera, semen abnormality parameters, and demographic parameters. Blue indicates a direct relationship while green shows an inverse relationship. *p < 0.05.

Generalized linear model analysis showed that *Lactobacillus* and *Collinsella* are positively correlated with sperm concentration and total sperm count. Moreover, *Collinsella* and *Bifidobacterium* were positively correlated with forward motile sperms. *Bacteroides*, *Prevotella*, and *Alicycliphilus* were negatively correlated with sperm concentration and total sperm count, and *Bacteroides* were positively correlated with age (Figure 8).

## 4. Discussion

This study investigated the relationship between the microbiota of the gut and urogenital tract and abnormalities in semen parameters. We found that the gut microbiota could be clustered into most OTUs, followed by urine and semen microbiota. The gut microbiota had a higher α-diversity and was significantly different from that of urine and semen. Furthermore, the β-diversity of the microbes was significantly different among the gut, semen, and urine. The composition of the urine and semen microbiota was similar but different from that of the gut (Figures 1B,D). These results are similar to those of a previous study (Lundy et al., 2021). Our study is the first showing that at the genus level, *Bifidobacterium*, *Blautia*, *Bacteroides*, *Faecalibacterium*, and *Prevotella* are predominant in the gut, *Staphylococcus*, *Streptococcus*, *Prevotella*, *Finegoldia*, and *Corynebacterium* are predominant in urine, and *Lactobacillus*, *Prevotella*, *Finegoldia*, *Staphylococcus*, *Streptococcus*, *Ureaplasma*, and other unidentified bacteria are predominant in semen (Figure 1D). A significant reduction in the semen abundance of *Staphylococcus* after vasectomy has also been reported (Lundy et al., 2021). Our study showed no significant differences in the α-diversity of the gut, semen, and urine microbiota between groups, which is consistent with previous reports (Baud et al., 2019; Garcia-Segura et al., 2022). However, few studies have reported differences in the α-diversity of semen microbes (Chen et al., 2018; Lundy et al., 2021; Yao et al., 2022). This might be due to differences in study participants as well as differences in their geographic location and the surrounding environment; however, this hypothesis needs to be further investigated.

Regarding the gut microbiota, our study showed that the abundance of *Collinsella* is significantly reduced in groups 1, 3, and 4 compared to that in the control group. This indicates that gut *Collinsella* has a beneficial effect on sperm motility, sperm count, and semen viscosity. Hirayama et al. (2021) showed that ursodeoxycholate produced by *Collinsella* prevents SARS-CoV-2 infection and improves acute respiratory distress syndrome in patients with COVID-19. Therefore, *Collinsella* not only exerts a beneficial effect on semen quality but also plays an important role in the control of COVID-19. The gut abundance of *Blautia* and *Bifidobacterium* was significantly reduced in the asthenospermia group compared to the control group. *Bifidobacterium* is a probiotic that protects against other harmful bacteria, improves gastrointestinal barrier function, inhibits pro-inflammatory cytokine production (Xue et al., 2017), improves memory functions (Azad et al., 2018), and alleviates food allergies (Fu et al., 2017). Supplementation with *Bifidobacterium* can improve sperm motility (Valcarce et al., 2017). It has been shown that cultured gut *Bifidobacterium* produces γ-aminobutyric acid (GABA) (Cai et al., 2022), which is important for fertilization. GABA not only promotes the acrosome reaction (Kurata et al., 2019) but also facilitates sperm capacitation by promoting tyrosine phosphorylation of the sperm proteins (Cai et al., 2022). *Blautia* can also inhibit pathogenic bacterial colonization in the gut by producing bacteriocins, which inhibit the proliferation of *Clostridium perfringens* and vancomycin-resistant enterococci (Liu et al., 2021). The oligospermatozoa group (Group_2) had a low gut abundance of differential bacteria, mainly *Clostridium sensu stricto 1*, the *Christensenellaceae* R-7 group, and *Intestinibacter*. Butyric acid is an important component of SCFAs. Butyrate plays an important role in male fertility by improving testicular endocrine functions and BTB permeability (Al-Asmakh et al., 2014). Supplementation with butyrate increases sperm count, sperm motility, and testosterone levels in adult roosters (Alhaj et al., 2018). *Blautia*, *Bifidobacterium*, *Lactobacillus*, and the *Christensenellaceae* R-7 group can produce SCFAs (Kim et al., 2014; Calderon-Perez et al., 2020; Markowiak-Kopec and Slizewska, 2020; Hajar-Azhari et al., 2021). *Blautia* could also prevent the development of obesity-related diseases (Kimura et al., 2013). *Intestinibacter* can reduce the risk of type I diabetes, making it a potential probiotic (Russell et al., 2019). The gut abundance of *Bacteroides* was significantly increased in Group_3, and the generalized linear model analysis showed that gut *Bacteroides* are positively correlated with age and negatively correlated with semen sperm concentration and total sperm count. A previous cohort study also showed a higher proportion of *Bacteroides* in the intestinal flora of older adults (Claesson et al., 2011). Our study suggests that *Bacteroides* are potentially pathogenic bacteria.

A significant increase in the abundance of *Staphylococcus* was observed in the semen of asthenospermia and seminal hyperviscosity only groups compared to the control group. Studies have shown that *Staphylococcus* may impair the secretory capacity of the prostate, seminal vesicles, and epididymis and may even reduce semen quality (Marconi et al., 2009). Consistent with our results, a previous study in bovines showed that an increase in the semen abundance of *Staphylococcus* reduces sperm viability and induces oxidative stress, leading to sperm DNA damage (Duracka et al., 2021). The OTUs that were differentially represented in Group_2 and Group_3 were very few, and mainly included *Bacillus* and *Enterobacter* (Figures 5B,C), whose abundance was increased and decreased, respectively. This indicates that semen flora has very little effect on sperm count.

*Lactobacillus* is a known probiotic that has positive effects on several physiological functions of the human body. Previous studies have shown that the presence of *Lactobacillus* in semen improves its quality (Weng et al., 2014; Monteiro et al., 2018; Baud et al., 2019; Alqawasmeh et al., 2022; Garcia-Segura et al., 2022) and boosts the success rate of assisted reproduction in infertile women (Moreno et al., 2022). Studies have also shown that supplementation with *Lactobacillus reuteri* can improve sperm motility (Valcarce et al., 2017) and increase testosterone levels in aged male mice (Poutahidis et al., 2014). Supplementation with *L. casei rhamnosus Döderleini* improves sperm immunogenicity and might be helpful for couples suffering from recurrent immunological spontaneous abortion (Rafiee et al., 2022). Oral administration of the three commensal *Lactobacillus* spp. can enhance sperm quality in dogs (Mahiddine et al., 2022). In addition, supplementation with *L. salivarius* CECT5713 and *L. gasseri* CECT5714 improves the symptoms of lactational mastitis caused by *Staphylococcus* in women (Jimenez et al., 2008). In summary, our study showed that the urinary abundance of *Lactobacillus* was significantly reduced in the oligospermia and seminal hyperviscosity only groups, suggesting that *Lactobacillus* could be a potential probiotic with beneficial effects on semen quality. In addition, the urinary abundance of the *Tissierellia* bacterium S7-1-4 was significantly reduced in the asthenospermia group.

Previous studies have shown that *Prevotella* is associated with abnormal semen parameters (Weng et al., 2014; Baud et al., 2019; Farahani et al., 2021). Similarly, generalized linear models in the present study also showed that Prevotella and Alicycliphilus are negatively correlated with sperm concentration and total sperm count. Moreover, we found that Lactobacillus and Collinsella are positively correlated with sperm concentration and total sperm count. Therefore, these microbiotas could be important factors for assessing semen quality.

However, our study has some limitations. This study was a single-center study with a small sample size. First, mid -stream urination and masturbation both contain urethral microbiomes, which are present in the urethra. Catheterization and seminal vesicle aspiration are more appropriate; however, catheterization and seminal vesicle aspiration are unlikely to be acceptable and appropriate for the volunteers. In moreover, neither negative control along the sampling process nor decontamination software were used to reduce potential contamination. Second, future multi-omics approaches such as metabolomics need to be combined to further elucidate the mechanisms of semen quality decline. Repeat sequencing rather than single sequencing can improve the accuracy of results. In addition, semen parameters such as sperm DNA damage, viability and mitochondrial membrane potential in relation to microbiota need to be explored in the future. Finally, fecal microbiota transplantation on experimental animals is needed to further validate the observations of this study, and studies with larger numbers of participants are needed to draw conclusions.

## 5. Conclusion

This study describes the flora of the intestinal and genitourinary tracts in healthy individuals and those with abnormal semen parameters. Our study suggests that *Collinsella*, *Bifidobacterium*, *Blautia,* and *Lactobacillus* can be used as probiotics. Furthermore, *Bacteroides* in the gut and *Staphylococcus* in semen are potentially pathogenic bacteria. The number and the proportion of differentially abundant bacteria were higher in the asthenospermia and seminal hyperviscosity only groups in all three kinds of samples analyzed. These results suggest that microbiota dysbiosis is closely associated with asthenospermia and seminal hyperviscosity. The differential abundance of OTUs between the healthy control and abnormal semen parameter groups was in the order of gut > semen > urine. This suggests that gut flora has the greatest influence on semen quality, followed by semen flora, while urinary flora plays a smaller role.

## Data availability statement

The datasets presented in this study can be found in online repositories. The names of the repository/repositories and accession number(s) can be found below: https://www.ncbi.nlm.nih.gov/, PRJNA900680.

## Ethics statement

The studies involving human participants were reviewed and approved by The Ethics Committee of Shandong First Medical University approved this study with ethics approval no. (R202203030053). The patients/participants provided their written informed consent to participate in this study.

## Author contributions

TC, SW, XL, and FG: experimental design. TC: experimental data collection and writing of the manuscript. BW, YZ, YW, and JT: semen analysis. TC, YP, SW, and QX: data analysis. XL: funded and revised the manuscript. All authors contributed to the article and approved the submitted version.

## Funding

This study was funded by National Natural Science Funds of China (82171594) and Zhao Yi-Cheng Medical Science Foundation (ZYYFY2018031).

## Conflict of interest

The authors declare that the research was conducted in the absence of any commercial or financial relationships that could be construed as a potential conflict of interest.

## Publisher’s note

All claims expressed in this article are solely those of the authors and do not necessarily represent those of their affiliated organizations, or those of the publisher, the editors and the reviewers. Any product that may be evaluated in this article, or claim that may be made by its manufacturer, is not guaranteed or endorsed by the publisher.

## Supplementary material

The Supplementary material for this article can be found online at: https://www.frontiersin.org/articles/10.3389/fmicb.2023.1182320/full#supplementary-material

Supplementary Figure S1 Barplot showing the phylum in the gut, urine, and semen.
